# Diaphragmatic Thickness as a Marker of Systemic Muscle Depletion in Non-Ambulatory Children with Cerebral Palsy: Implications for Integrated Neurorehabilitation Assessment

**DOI:** 10.3390/neurosci7050096

**Published:** 2026-08-31

**Authors:** Maracy Balbino Morgado Sobreira, Tatielle de Lima Vieira, Helga Cecília Muniz de Souza, Bárbara Bernardo Figueirêdo, Paulo Adriano Schwingel, Paulo André Freire Magalhães

**Affiliations:** 1 Graduate Program in Rehabilitation and Functional Performance (PPGRDF), Universidade de Pernambuco (UPE), Petrolina 56328-900, PE, Brazil; maracy.sobreira@upe.br (M.B.M.S.); tatielle.lvieira@upe.br (T.d.L.V.); barbara.bernardo@upe.br (B.B.F.); paulo.schwingel@upe.br (P.A.S.); 2 Research Group in Neonatology and Pediatrics (Baby GrUPE), Universidade de Pernambuco (UPE), Petrolina 56328-900, PE, Brazil; 3 Laboratório de Pesquisas em Desempenho Humano (LAPEDH), Universidade de Pernambuco (UPE), Petrolina 56328-900, PE, Brazil; 4 Hospital das Clínicas, Federal University of Pernambuco (HC-UFPE), Recife 50670-901, PE, Brazil; helgamuniz@yahoo.com.br

**Keywords:** cerebral palsy, diaphragmatic ultrasonography, arm muscle area, Gross Motor Function Classification System, pediatric rehabilitation, respiratory muscle assessment, nutritional status

## Abstract

Purpose: To examine the association between diaphragmatic thickness and upper arm muscle area (AMA), as a surrogate of nutritional status, in children and adolescents with cerebral palsy (CP) stratified by gross motor function severity. Methods: In this cross-sectional study, participants were stratified into ambulatory (Gross Motor Function Classification System [GMFCS] I–III) and non-ambulatory (GMFCS IV–V) groups. AMA was estimated from anthropometric measurements, and diaphragmatic thickness was assessed by B- and M-mode ultrasonography; between-group comparisons used the Mann–Whitney U test and associations were examined using Spearman’s rank correlation coefficient (ρ). Results: Fifty children aged 2–12 years with a confirmed CP diagnosis were evaluated (14 ambulatory, 36 non-ambulatory). In the non-ambulatory group, diaphragmatic thickness showed significant positive correlations with AMA during inspiration (ρ = 0.50; *p* = 0.002) and expiration (ρ = 0.67; *p* < 0.001). These subgroup-specific associations should be interpreted as exploratory, as the AMA × GMFCS interaction did not reach statistical significance. No significant correlations were observed in ambulatory participants. Moderate muscle mass deficits were prevalent among non-ambulatory children, and approximately 63% of the total sample presented adipose tissue deficit or risk thereof. Conclusions: In children with severe CP, a significant association between peripheral muscle reserve and diaphragmatic thickness suggests a possible functional link between nutritional status and respiratory muscle morphology. These preliminary findings support further investigation of diaphragmatic ultrasonography as a non-invasive tool for combined nutritional and respiratory monitoring in pediatric neurorehabilitation.

## 1. Introduction

Cerebral palsy (CP) refers to a group of permanent conditions affecting movement, posture, and motor function (encompassing spasticity, dystonia, choreoathetosis, and/or ataxia) that arise from a non-progressive disturbance in the developing fetal or infant brain [1,2]. CP is increasingly recognized as a lifelong neurodevelopmental condition associated with primary and secondary impairments that evolve across the lifespan [2], and constitutes the leading cause of childhood physical disability, with a global pooled prevalence of approximately 2.11 per 1000 live births [3]. These impairments precipitate a cascade of secondary musculoskeletal and systemic complications that evolve over time [4,5]. Among these, respiratory compromise is a primary determinant of morbidity and mortality in this population [1,3].

The etiology of respiratory failure in CP is multifactorial, arising from a confluence of impaired airway clearance due to bulbar dysfunction and recurrent aspiration, chronic airway obstruction, and compromised ventilatory pump function [1,5]. The latter is often exacerbated by progressive musculoskeletal deformities, such as scoliosis and thoracic cage distortions, which impose severe restrictive constraints on lung mechanics and impair efficient gas exchange [4,5].

Central to this respiratory compromise is the dysfunction of the ventilatory muscles. Despite its profound clinical impact, respiratory muscle weakness remains an underappreciated and frequently underdiagnosed comorbidity in routine clinical practice for CP [1,6]. As the principal muscle of inspiration, the diaphragm is particularly susceptible to the effects of chronic disease, including disuse atrophy secondary to mechanical ventilation or sedentary behavior, altered central neuromuscular activation, and increased mechanical load imposed by chest wall rigidity—conditions especially prevalent in individuals with severe motor impairment [6,7].

Concurrently, peripheral skeletal muscle wasting is a hallmark of the CP phenotype, profoundly affecting both appendicular and axial musculature. This systemic muscle depletion contributes directly to diminished mobility, heightened energy expenditure during activities of daily living, and reduced functional independence [8,9]. This process may be accelerated by nutritional deficits, which are associated with loss of lean body mass and a potential cycle of functional deterioration and clinical decline [7,10].

Traditional anthropometric measures, such as weight-for-age and body mass index (BMI), are often confounded by growth failure and postural abnormalities in children with CP, limiting their sensitivity for detecting early sarcopenia [11]. In contrast, the mid-upper arm muscle area (AMA), calculated from arm circumference and tricep skinfold thickness, serves as a robust and validated surrogate for total body muscle mass in pediatric populations with neuromuscular disorders [12,13]. In parallel, musculoskeletal ultrasonography has emerged as a non-invasive, reliable, and sensitive tool for quantifying the thickness and contractility of the diaphragm, enabling the objective assessment of muscle atrophy, hypertrophy, or structural changes resulting from chronic respiratory loading [6,14,15].

Although the link between systemic nutritional status and global muscle integrity is well-established, the specific relationship between peripheral somatic muscle mass and respiratory muscle structure in CP remains largely uninvestigated [14,15,16]. Prior work from our group has characterized diaphragmatic function in children with CP using ultrasonography [17]; however, the specific association between peripheral muscle reserves and diaphragmatic morphology, stratified by motor severity, remains unestablished. This knowledge gap is clinically significant, as compromised nutritional status and peripheral sarcopenia may deplete the diaphragm’s metabolic and structural reserves, thereby limiting ventilatory capacity and increasing susceptibility to pulmonary complications [1,6,8,9].

Therefore, this study was designed to address this critical gap in the literature. The primary objective was to examine the association between diaphragmatic thickness, assessed by ultrasonography, and AMA, as a surrogate of peripheral muscle reserves and nutritional status, in children and adolescents with CP according to gross motor function severity. We hypothesized that diminished diaphragmatic thickness would be associated with reduced AMA and explored whether the magnitude of this association differed according to GMFCS group. Because the study was cross-sectional and subgroup differences may not establish effect modification, these analyses were considered exploratory. Characterizing this relationship may contribute to the development of integrated and individualized assessment protocols in pediatric neurorehabilitation, particularly for children with severe motor impairment.

## 2. Materials and Methods

### 2.1. Study Design

This observational, cross-sectional study was conducted and reported in accordance with the Strengthening the Reporting of Observational Studies in Epidemiology (STROBE) guidelines [18]. The study protocol received ethical approval from the Research Ethics Committee of the Centro Integrado de Saúde Amaury de Medeiros da Universidade de Pernambuco (CISAM/UPE), under certificate of presentation for ethical appraisal (CAAE) number 60569722.6.0000.5191. The investigation conformed to the principles outlined in the Declaration of Helsinki and complied with Brazilian National Health Council Resolution No. 466/2012, which governs research involving human subjects in Brazil. Written informed consent was obtained from the parents or legal guardians of all participants prior to any study-related procedures.

### 2.2. Participants and Setting

The inclusion criteria were: (a) a formal medical diagnosis of non-progressive neurological injury consistent with CP; (b) classification across all functional levels of the Gross Motor Function Classification System (GMFCS) from level I to level V; and (c) clinical stability, defined as an absence of an acute respiratory infection or an exacerbation of chronic conditions within two weeks prior to the assessment.

The exclusion criteria were: (a) acute hemodynamic instability; (b) use of neuromuscular blocking agents; (c) a history of major thoracic or spinal surgery (e.g., scoliosis correction) that could directly alter chest wall mechanics; or (d) the presence of chest drains or non-invasive ventilation at the time of data collection.

Children with untreated or non-surgically managed chest-wall deformity of any degree were not excluded, as this is intrinsic to the severe CP phenotype; excluding these children would have produced a non-representative sample and undermined the external validity of a study focused on this population. Only children with a history of major thoracic or spinal surgery (which would directly and mechanically alter chest-wall biomechanics) were excluded (exclusion criterion c).

### 2.3. Gross Motor Function Classification

To investigate the influence of the severity of motor impairment, the participants were a priori stratified into two subgroups based on functional mobility: Group 1, with less severe impairment (GMFCS levels I–III), comprised individuals with ambulatory potential, and Group 2, with more severe impairment (GMFCS levels IV–V), comprised non-ambulatory individuals who typically require wheeled mobility for self-propulsion.

The classification of each participant was confirmed by reviewing recent medical or physiotherapy records provided by the child’s primary healthcare team to ensure alignment with established GMFCS guidelines [19,20,21].

### 2.4. Standardized Assessment Protocol

To ensure high methodological rigor and minimize potential sources of measurement error, all assessments were conducted within a single session (approximately 45–60 min) in a dedicated laboratory space under strictly controlled environmental conditions. The ambient temperature was maintained at 22–24 °C, relative humidity at 40–60%, and ambient noise levels were kept to a minimum. Standardized, adjustable lighting was used to ensure optimal visibility for all measurements while avoiding participant discomfort.

Prior to data collection, each participant underwent a 10 min acclimatization period to the environment. This step was crucial to establish stable cardiorespiratory baselines and mitigate potential artifacts arising from stress or anxiety. To promote participant comfort and maximize cooperation, a parent or legal guardian was present throughout the entire procedure. To eliminate inter-rater variability, all anthropometric and ultrasonographic measurements were performed by the same team of trained investigators following a standardized operational protocol.

### 2.5. Anthropometric Measurements

Body weight was measured to the nearest 0.1 kg using a calibrated digital scale model AVA-340 (Avanutri Equipamentos de Saúde Ltd.a., Três Rios, RJ, Brazil). For non-ambulatory children, weight was determined by subtraction: the caregiver was first weighed alone, then weighed again while holding the child, and the difference was recorded. For ambulatory participants (GMFCS I–III), stature was measured directly using a stadiometer. For non-ambulatory participants (GMFCS IV–V), stature was estimated from knee height, measured using an inelastic tape from the heel to the anterior surface of the thigh with the knee and ankle flexed at 90° and the child positioned supine. The Stevenson equation [11] was then applied to convert knee height into an estimated total body height.

The mid-upper arm circumference (MUAC) was measured at the midpoint between the acromion and olecranon processes on the less affected arm. Tricep skinfold thickness (TSF) was measured in millimeters at the same site using a calibrated digital skinfold caliper (Avanutri Equipamentos de Saúde Ltd.a.). Two consecutive measurements were obtained at each site; when values differed, a third measurement was performed, and the mean of the two closest values was used as the final result.

AMA and arm fat area (AFA) were calculated from these primary measures using the following standard equations [12,14]:

- AMA (cm^2^) = [(MUAC − (π × TSF))^2^]/(4π).
- AFA (cm^2^) = [MUAC^2^/(4π)] − AMA.

These derived indices serve as validated surrogates for peripheral muscle and fat stores, and were used to characterize the nutritional status of the participants [13].

### 2.6. Diaphragmatic Thickness Measurement

A Logiq E ultrasound system (GE Medical Systems, Wuxi, China) equipped with a high-frequency linear transducer (7.5–12 MHz) was used to perform diaphragmatic ultrasound. To eliminate inter-operator variability, a single highly experienced physical therapist with over a decade of expertise in pediatric respiratory ultrasonography conducted all examinations.

Participants were positioned in a semi-recumbent posture with a 45° inclination and were instructed to breathe quietly and spontaneously. The transducer was placed perpendicular to the chest wall in the zone of apposition, typically between the eighth and eleventh intercostal spaces along the right anterior axillary line, to acquire a clear acoustic window of the right hemidiaphragm. The diaphragm appeared as a three-layered structure: a central non-echogenic muscle layer bordered by two hyperechoic lines representing the diaphragmatic pleura and peritoneum.

The primary outcome measures were diaphragmatic thickness at end-expiration (Tdi,ee) and at end-inspiration (Tdi,ei). After using B-mode to anatomically localize the area, measurements were taken in M-mode at the point of maximal relaxation during the tidal breathing cycle. Diaphragmatic thickness was defined as the vertical distance between the inner edges of the pleural and peritoneal lines. To reduce intra-subject variability, the final value was calculated as the mean of three separate measurements from three consecutive, stable respiratory cycles. All acquired images and video loops were digitally stored for offline verification and quality control.

Intra-session measurement consistency was assessed from these triplicate measurements: reliability was excellent for expiratory thickness (ICC = 0.997 for the averaged triplicate) and good for inspiratory thickness (ICC = 0.79 for the averaged triplicate; ICC = 0.55 for a single measurement), with a mean within-subject coefficient of variation of approximately 15% for both measures. This intra-session estimate reflects same-visit, same-examiner consistency rather than separate-day intra-rater or multi-examiner inter-rater reliability, which were not assessed in this study.

### 2.7. Statistical Analysis

No formal a priori sample size calculation was performed for this study. All data collected were anonymized and analyzed using IBM^®^ SPSS^®^ Statistics (IBM Corp., Armonk, NY, USA, release 22.0, 2013). Figures were generated using GraphPad Prism (GraphPad Software, San Diego, CA, USA, release 10.1.2, 2023). The Shapiro–Wilk test confirmed non-normal distribution for all continuous variables (*p* < 0.05); therefore, descriptive data are presented as medians with first quartile (1Q) and third quartile (3Q). Categorical data, such as GMFCS subgroups, were presented as absolute frequencies (n) and relative frequencies (%), and these were compared using Fisher’s exact test. Differences in anthropometric and diaphragmatic variables between the two GMFCS subgroups (GMFCS I–III vs. GMFCS IV–V) were assessed using the Mann–Whitney *U* test for independent samples. Additionally, the primary analysis investigated the monotonic relationship between diaphragmatic thickness (Tdi,ee and Tdi,ei) and AMA using Spearman’s rank correlation coefficient (ρ). Subgroup analyses were performed to assess this correlation separately within the GMFCS I–III and GMFCS IV–V groups. To address the observed age difference between GMFCS subgroups, partial Spearman correlations between AMA and diaphragmatic thickness were computed controlling for age, and a multivariable linear regression was fitted with diaphragmatic thickness as the dependent variable and AMA, age, sex, and GMFCS group as independent variables (*n* = 50; 4 predictors; subjects-per-variable = 12.5).

All statistical tests were two-tailed, with exact *p*-values reported throughout. Statistical significance was defined as *p* ≤ 0.05. Given the six pre-specified primary Spearman correlations (total cohort, ambulatory, and non-ambulatory strata, each for inspiratory and expiratory thickness), a Bonferroni-corrected significance threshold (α = 0.05/6 = 0.0083) was applied as a sensitivity analysis. Rank-biserial correlation (*r*) was used to estimate the magnitude of between-group differences for all Mann–Whitney comparisons, categorized per conventional benchmarks (0.10: small; 0.30: medium; 0.50: large) [22,23]. Correlation strength was interpreted according to Schober, Boer & Schwarte [24]: negligible <0.10; weak 0.10–0.39; moderate 0.40–0.69; strong 0.70–0.89; very strong 0.90–1.00.

## 3. Results

### 3.1. Participant Characteristics

**Figure 1:**
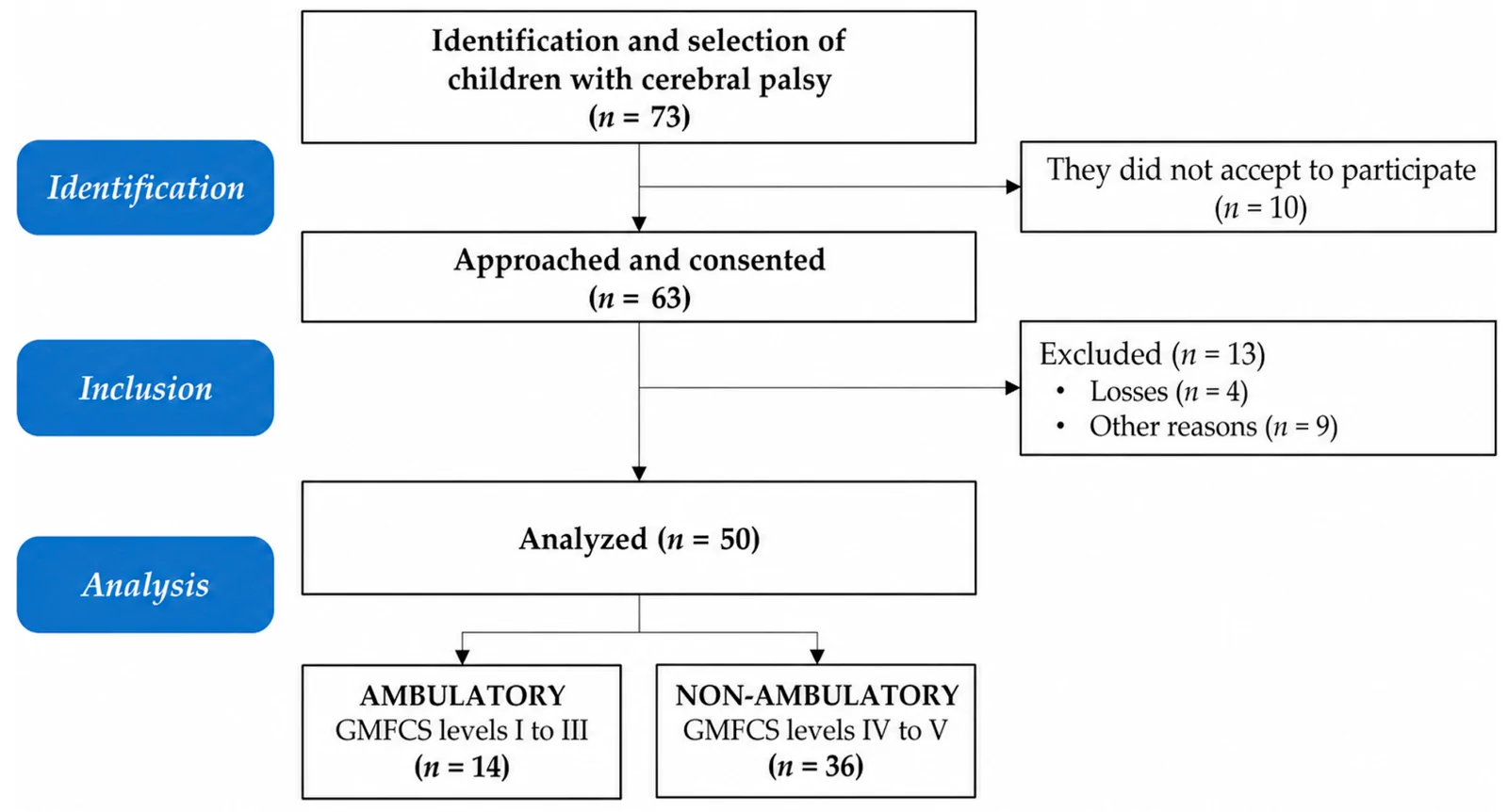
Study participant flow diagram. GMFCS: Gross Motor Function Classification System.

A total of 50 children and adolescents with CP (58% male) with a median (1Q–3Q) age of 7.3 years (5.1–10.1 years) were included in the study (Figure 1). According to the GMFCS, 14 participants (28%) were classified as ambulatory (GMFCS levels I–III), while 36 (72%) were non-ambulatory (GMFCS levels IV–V).

The baseline anthropometric and clinical characteristics of the participants, stratified by GMFCS group, are presented in Table 1. The non-ambulatory group was significantly older than the ambulatory group (median 8.7 vs. 5.6 years, respectively; *p* = 0.016). No other significant between-group differences were observed for any anthropometric variable, including total body mass, height, BMI, AMA, or AFA (*p* > 0.05). Regarding nutritional status, moderate-to-severe muscle mass deficits were more prevalent in the non-ambulatory group, with approximately 44% classified with severe deficit (AMA ≤ 5th percentile). For adipose tissue reserves, approximately 63% of the total sample presented with adipose tissue deficit or risk thereof (AFA ≤ 15th percentile), with proportions distributed similarly across both GMFCS groups (*p* > 0.05).

No significant sex differences were observed for AMA (male median 16.08 vs. female 16.35 cm^2^, *p* = 0.93) or diaphragmatic thickness (inspiratory: 1.27 vs. 1.30 mm, *p* = 0.11; expiratory: 0.63 vs. 0.67 mm, *p* = 0.34). Chest-wall configuration was recorded for every participant (Table 2). None of the 14 ambulatory participants had a kyphoscoliotic or funnel chest, whereas 9/36 (25.0%) non-ambulatory participants did (7 kyphoscoliotic, 2 infundibiliform)—a significantly higher prevalence of chest-wall deformity in the non-ambulatory group (*p* = 0.047). No participant in either group had a documented history of surgical chest/spine correction.

Similarly, expiratory and inspiratory diaphragmatic thickness did not differ significantly between the GMFCS groups (Table 3).

### 3.2. Muscle Mass and Diaphragmatic Thickness

**Figure 2:**
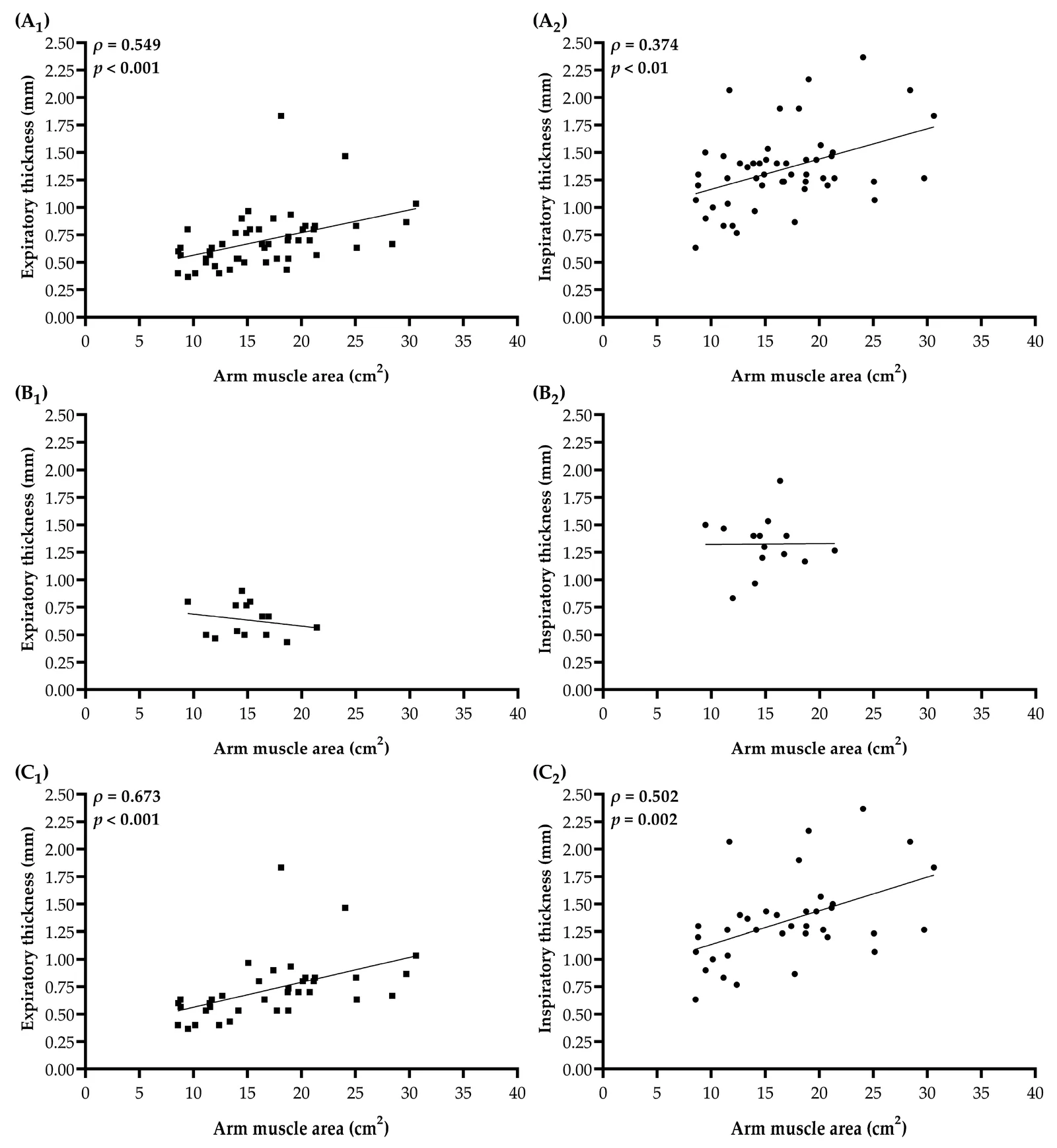
Correlation between arm muscle area (AMA) and diaphragmatic thickness in children and adolescents with cerebral palsy, stratified by motor function. Scatterplots illustrate the relationship between AMA and both expiratory and inspiratory diaphragmatic thickness. The analysis is presented for the total cohort (Panels (**A**)) and stratified into ambulatory (GMFCS I–III; Panels (**B**)) and non-ambulatory (GMFCS IV–V; Panels (**C**)) subgroups. Column 1 depicts the correlation with expiratory thickness, while column 2 depicts the correlation with inspiratory thickness.

The primary analysis examined the relationship between peripheral muscle mass (AMA) and diaphragmatic thickness. Figure 2 illustrates the results of the Spearman correlation analyses for the total cohort and for the GMFCS subgroups.

Overall, a significant, moderate positive correlation was found between AMA and both expiratory (ρ = 0.544; *p* < 0.001) and inspiratory (ρ = 0.374; *p* = 0.01, not significant after Bonferroni correction for the six primary correlations tested [α = 0.0083]) thickness.

When described according to motor function, the association was statistically significant in the non-ambulatory group (GMFCS IV–V), but not in the ambulatory group. In the non-ambulatory subgroup, AMA showed a moderate-to-strong positive correlation with expiratory thickness (ρ = 0.673; *p* < 0.001) and a moderate positive correlation with inspiratory thickness (ρ = 0.502; *p* = 0.002); both remained significant after Bonferroni correction. However, because the AMA × GMFCS interaction term did not reach statistical significance, these subgroup-specific findings should be interpreted cautiously as exploratory and hypothesis-generating rather than as evidence that GMFCS significantly modified the association. In contrast, no significant correlation was found between AMA and diaphragmatic thickness in the ambulatory group (GMFCS I–III) for expiratory (Figure 2, Panel B_1_) or inspiratory (Figure 2, Panel B_2_) measurements (*p* > 0.05).

### 3.3. Sensitivity Analyses: Partial Correlation and Multivariable Regression

The AMA–diaphragm association remained statistically significant after controlling for age (Table S1). In the non-ambulatory subgroup, the partial Spearman correlation adjusted for age was ρ = 0.562 (*p* < 0.001) for expiratory thickness and ρ = 0.408 (*p* = 0.015) for inspiratory thickness (zero-order ρ = 0.672 and ρ = 0.494, respectively). In multivariable linear regression (Table S2; expiratory thickness as the dependent variable; AMA, age, sex, GMFCS group, and the AMA × GMFCS interaction term as independent variables), AMA remained associated with expiratory diaphragmatic thickness (B = 0.017 mm/cm^2^, 95% confidence interval: 0.003–0.031, *p* = 0.018). The AMA × GMFCS interaction term was not statistically significant, indicating that the available data did not provide statistical evidence that GMFCS modified the AMA–diaphragm association. These findings support an association between AMA and diaphragmatic thickness after adjustment for age, but do not establish GMFCS-dependent effect modification.

## 4. Discussion

This cross-sectional study investigated the association between peripheral muscle reserves, estimated via AMA, and diaphragmatic thickness in children and adolescents with CP. Our primary finding was a moderate-to-strong positive correlation between AMA and expiratory diaphragmatic thickness in non-ambulatory participants (GMFCS IV–V; ρ = 0.67), whereas no statistically significant correlation was observed in the ambulatory subgroup. However, the AMA × GMFCS interaction term did not reach statistical significance. Therefore, the difference in subgroup-specific estimates should be interpreted cautiously as an exploratory and hypothesis-generating pattern rather than as confirmed evidence of GMFCS-dependent effect modification. These findings are compatible with a possible association between peripheral muscle reserves and respiratory muscle morphology in children with severe CP, but they do not establish a differential relationship according to motor severity [1,5,8,10].

An intriguing aspect of our results is that, although absolute diaphragmatic thickness did not significantly differ between groups, the subgroup-specific estimates of the association between diaphragmatic thickness and peripheral muscle mass differed descriptively. Because the AMA × GMFCS interaction term was not statistically significant, these differences should not be interpreted as evidence that the underlying pathophysiology differs definitively according to motor impairment. In the ambulatory group (GMFCS I–III), no statistically significant correlation was observed between diaphragmatic thickness and AMA; however, the small subgroup size limits the ability to conclude that no association exists. Higher daily activity and less severe postural deformities may contribute to preservation of diaphragmatic structure, but this possibility requires confirmation in larger and longitudinal studies [7,25].

In the non-ambulatory group, the moderate-to-strong correlation between AMA and expiratory diaphragmatic thickness is compatible with the possibility of a shared relationship between peripheral and respiratory muscle status. However, because the AMA × GMFCS interaction term was not statistically significant, severe motor impairment should not be described as a confirmed moderator of this association. In these children—who were also significantly older in our cohort (*p* = 0.016); prolonged immobility, prevalent spasticity, chest-wall deformity (present in 25.0% of the non-ambulatory subgroup vs. none of the ambulatory subgroup in our sample, *p* = 0.047), and compromised nutritional intake may contribute to the depletion of peripheral and respiratory muscle reserves [8,9,26]. Diaphragmatic morphology may, in this context, reflect systemic processes that also affect limb muscle; however, evidence from critically ill adults and preclinical models cannot be directly extrapolated to children with CP [27,28]. This represents a plausible but unconfirmed mechanism that should be investigated in future CP-specific studies [29,30].

These findings underscore the critical limitations of using traditional anthropometric markers like BMI to guide clinical decisions in this population. Our data aligned with literature showing that BMI can mask significant sarcopenia due to altered body composition and linear growth failure in CP [11,31,32]. The combined use of a simple, field-applicable measure like AMA (validated for assessing nutritional status [12,13]) and a non-invasive imaging tool like bedside ultrasonography provides a far more granular and clinically relevant assessment [14,33]. This dual approach allows for the simultaneous evaluation of systemic nutritional reserves and a key respiratory muscle, offering a more complete picture of the child’s functional vulnerability.

From a clinical and rehabilitation perspective, our findings support consideration of integrated assessment protocols for children with CP, including those classified as GMFCS IV–V. It is plausible that treating nutritional deficits with dietary supplementation alone may be insufficient if diaphragmatic integrity is concurrently compromised; a hypothesis that warrants prospective interventional testing. The moderate-to-strong association between AMA and diaphragm thickness in this subgroup suggests that interventions must be multimodal. This reinforces the growing body of evidence supporting targeted inspiratory muscle training (IMT), neuromuscular electrical stimulation, and functional physiotherapy to preserve respiratory function alongside nutritional rehabilitation in children with severe neuromotor disorders [7,34,35]. Recent systematic reviews and randomized trials indicate that IMT and feedback-device-based training can meaningfully increase inspiratory muscle strength and, for some protocols, ventilatory parameters in children and adults with CP [36,37,38], supporting the plausibility (though not, given our cross-sectional design, proof) that interventions preserving or improving peripheral and diaphragmatic muscle mass could translate structural preservation into functional respiratory benefit, particularly in the GMFCS IV–V subgroup.

### Limitations

This study has several limitations that should be acknowledged. Its cross-sectional design precludes the establishment of causality; findings are consistent with a possible association between systemic muscle depletion and diaphragmatic morphology, and longitudinal studies are necessary to determine whether a decline in AMA precedes, follows, or occurs in parallel with diaphragm thinning.

No formal a priori sample size calculation was performed for this study; the sample was determined pragmatically by the number of eligible, consenting children with a confirmed CP diagnosis accessible during the study period—a common constraint when recruiting non-ambulatory children with severe motor impairment, a population that is inherently difficult to access in the numbers required for a priori calculations targeting a pre-specified effect size. A post hoc sensitivity analysis indicates that the non-ambulatory subgroup (*n* = 36), in which our primary findings were observed, had 99.7% power to detect the observed expiratory correlation (ρ = 0.673) and 89.4% power to detect the observed inspiratory correlation (ρ = 0.502) at α = 0.05 (two-tailed), indicating that these central findings were not underpowered. The ambulatory subgroup (*n* = 14), by contrast, was powered to detect only correlations ≥ 0.68 (80% power, α = 0.05), and the absence of a significant correlation in that stratum should be interpreted in light of this constraint rather than as definitive evidence of no association.

The GMFCS IV–V group was significantly older than the GMFCS I–III group (*p* = 0.016); we addressed this directly via partial Spearman correlation and multivariable linear regression controlling for age, and the AMA–diaphragm association remained statistically significant net of age in both analyses, indicating that age does not fully account for the observed relationship. Residual confounding by unmeasured growth-related factors cannot, however, be entirely excluded, and prospective designs with repeated within-subject measures across development would further strengthen causal inference.

While the use of a single, highly experienced sonographer eliminated inter-rater variability during data collection, diaphragm ultrasound remains inherently operator-dependent [16]. We were able to compute intra-session measurement consistency from the triplicate measurements retained for each participant (excellent for expiratory thickness, ICC = 0.997; good for the averaged inspiratory measurement, ICC = 0.79); however, true separate-day intra-rater reliability and multi-examiner inter-rater reliability were not assessed and remain a priority for future validation studies.

Chest-wall deformity was recorded categorically (present in 25.0% of the non-ambulatory subgroup vs. none of the ambulatory subgroup, *p* = 0.047) but not graded using a quantitative radiographic index such as Cobb angle, limiting our ability to model its independent contribution to diaphragmatic morphology; future studies should incorporate standardized radiographic grading of chest-wall/spinal deformity.

Additionally, both AMA and diaphragmatic thickness are surrogate measures; future studies incorporating criterion-referenced methods such as dual-energy X-ray absorptiometry (DXA) or magnetic resonance imaging would further validate these associations. Future multi-center studies should prioritize a priori sample size calculations, standardized protocols, and formal inter-rater reliability assessment to enhance generalizability.

These findings suggest that the combination of segmental anthropometry and diaphragmatic ultrasound may be a feasible approach for investigating peripheral and respiratory muscle status in children with CP [14,17]. This integrated assessment may eventually help inform clinical risk stratification and individualized rehabilitation planning, but its prognostic and treatment-related value remains to be established. Before these assessments can be recommended for routine neurorehabilitation practice, prospective studies evaluating their predictive validity, responsiveness to intervention, reproducibility, and association with clinically relevant respiratory outcomes are needed.

## 5. Conclusions

This study reveals a significant positive correlation between peripheral muscle mass and diaphragmatic thickness exclusively in children and adolescents with severe CP (GMFCS IV–V), with no such association observed in those with milder motor impairment, and this association remained statistically significant after adjusting for age. This GMFCS-dependent dichotomy is consistent with a possible role of motor severity as a moderator of systemic muscle depletion, which may be associated with changes in both limb and respiratory musculature in this population.

These findings support the combined use of bedside diaphragmatic ultrasonography and segmental anthropometry as a promising strategy for identifying systemic muscle depletion in children with severe CP, with potential to inform targeted, individualized nutritional and respiratory interventions pending confirmation in prospective, outcome-linked studies.

## Figures and Tables

**Table 1:** Baseline anthropometric and clinical characteristics of children and adolescents with cerebral palsy, stratified by Gross Motor Function Classification System (GMFCS) group.

VARIABLES	TOTAL *n* = 50	GROUPS BASED ON GMFCS		*p* ^1^	*r*
		AMBULATORY (Levels I to III)	NON-AMBULATORY (Levels IV to V)		
		*n* = 14	*n* = 36		
	Median (1Q–3Q)	Median (1Q–3Q)	Median (1Q–3Q)		
Age, years	7.3 (5.1–10.1)	5.6 (5.1–7.0)	8.7 (5.4–10.8)	0.016	0.33
Total body mass, kg	18.5 (13.0–23.5)	18.1 (12.5–22.1)	19.1 (13.2–24.1)	0.482	0.04
Upper arm muscle area, cm^2^	16.2 (11.9–19.8)	14.8 (13.4–16.8)	17.6 (11.7–20.6)	0.295	0.09
Upper arm fat area, cm^2^	4.3 (3.4–7.4)	4.1 (3.5–7.4)	4.4 (3.4–7.6)	0.804	0.02
Height, cm	115.7 (98.6–127.9)	110.0 (106.7–116.3)	120.8 (98.6–128.9)	0.157	0.14
Tricep skinfold thickness, mm	5.7 (4.5–9.2)	5.6 (4.5–9.2)	5.9 (4.5–9.2)	0.837	0.02
Upper arm circumference, cm	16.4 (14.4–18.0)	16.0 (14.8–17.2)	16.8 (14.4–18.0)	0.398	0.06
Body mass index (BMI), kg/m^2^	14.6 (13.0–16.1)	15.0 (14.2–16.3)	14.3 (12.7–16.1)	0.310	0.19
Chest-wall deformity, *n* (%) *	9 (18.0%)	0 (0.0%)	9 (25.0%)	0.047	–

1Q: first quartile; 3Q: third quartile; *r*: rank-biserial. * Values are presented as total frequency and percentage. ^1^ All *p*-values are from the Mann–Whitney *U* test except for chest-wall deformity (Fisher’s exact test); rank-biserial *r* is not applicable to this categorical comparison.

**Table 2:** Chest-wall configuration by Gross Motor Function Classification System (GMFCS) group.

CHEST-WALL CONFIGURATION	GROUPS BASED ON GMFCS		*p* ^1^
	AMBULATORY (Levels I to III)	NON-AMBULATORY (Levels IV to V)	
	*n* = 14	*n* = 36	
	*n* (%)	*n* (%)	
Normal	14 (100.0%)	27 (75.0%)	0.047
Funnel (infundibiliform)	0 (0.0%)	2 (5.6%)	
Kyphoscoliotic	0 (0.0%)	7 (19.4%)	

^1^ *p* = 0.047, Fisher’s exact test comparing any deformity versus normal.

**Table 3:** Comparison of inspiratory and expiratory diaphragmatic thickness in children and adolescents with cerebral palsy, stratified by the Gross Motor Function Classification System (GMFCS) group.

VARIABLES	TOTAL *n* = 50	GROUPS BASED ON GMFCS		*p* ^1^	*r*
		AMBULATORY (Levels I to III)	NON-AMBULATORY (Levels IV to V)		
		*n* = 14	*n* = 36		
	Median (1Q–3Q)	Median (1Q–3Q)	Median (1Q–3Q)		
Inspiratory thickness, mm	1.30 (1.19–1.47)	1.35 (1.19–1.48)	1.28 (1.10–1.46)	0.863	0.02
Expiratory thickness, mm	0.67 (0.53–0.80)	0.62 (0.50–0.78)	0.67 (0.54–0.83)	0.289	0.15

1Q: first quartile; 3Q: third quartile; *r*: rank-biserial. ^1^ *p*-value from the Mann–Whitney *U* test.

## Data Availability

The datasets generated during and/or analyzed during the current study are not publicly available due to the presence of sensitive or confidential patient information but are available from the corresponding authors on reasonable request.

## Supplementary Materials

The following supporting information can be downloaded at: https://www.mdpi.com/article/10.3390/neurosci7050096/s1, Table S1: partial Spearman correlation between arm muscle area (AMA) and diaphragmatic thickness in children and adolescents with cerebral palsy, adjusted for age; Table S2: multivariable linear regression for diaphragmatic thickness in children and adolescents with cerebral palsy, with arm muscle area (AMA), age, sex, and Gross Motor Function Classification System (GMFCS) group as independent variables.

## Abbreviations

The following abbreviations are used in this manuscript:

AFA	Arm fat area
AMA	Arm muscle area
BMI	Body mass index
CAAE	Certificate of presentation for ethical appraisal
CAPES	Coordination for the Improvement of Higher Education Personnel
CEP	Research Ethics Committee
CNPq	National Council for Scientific and Technological Development
CP	Cerebral palsy
DXA	Dual-energy X-ray absorptiometry
ES	Effect size
FACEPE	Fundação de Amparo à Ciência e Tecnologia do Estado de Pernambuco
GMFCS	Gross Motor Function Classification System
IMT	Inspiratory muscle training
MUAC	Mid-upper arm circumference
STROBE	Strengthening the Reporting of Observational Studies in Epidemiology
Tdi,ee	Diaphragmatic thickness at end-expiration
Tdi,ei	Diaphragmatic thickness at end-inspiration
TSF	Tricep skinfold thickness
