# Supplementary material for: Diaphragmatic Thickness as a Marker of Systemic Muscle Depletion in Non-Ambulatory Children with Cerebral Palsy: Implications for Integrated Neurorehabilitation Assessment
Source: NeuroSci. 2026 Aug 31;7(5):96. doi: 10.3390/neurosci7050096 (PMC13600196; doi:10.3390/neurosci7050096)
Supplement: Supplementary file 1 [file neurosci-07-00096-s001.zip › neurosci-4394174-supplementary.pdf]

Table S1. Partial Spearman correlation between arm muscle area (AMA) and diaphragmatic thickness in children and adolescents with cerebral palsy, adjusted for age.

**Outcome**	**Partial correlation (ϱ)**	***p***
Inspiratory thickness, mm	0.316	0.027
Expiratory thickness, mm	0.445	0.001

n = 50; *df* = 47 (controlling for 1 variable). Genuine partial Spearman correlation, computed by applying Pearson's partial correlation to the ranks (not the raw values) of AMA, diaphragmatic thickness, and age.

Table S2. Multivariable linear regression for diaphragmatic thickness in children and adolescents with cerebral palsy, with arm muscle area (AMA), age, sex, and Gross Motor Function Classification System (GMFCS) group as independent variables.

**Outcome**	**Predictor**	***B***	**95%CI**	***p***
Inspiratory thickness, mm	AMA	0.025	0,006–0,044	0,011
	Age	0.008	-0,026–0,043	0,635
	Sex	0.180	-0,017–0,376	0,072
	GMFCS	-0.011	-0,232–0,211	0,924
Expiratory thickness, mm	AMA	0.017	0,003–0,031	0,018
	Age	0.011	-0,014–0,037	0,362
	Sex	0.055	-0,089–0,198	0,448
	GMFCS	0.033	-0,129–0,194	0,684

B: unstandardized regression coefficient; 95%CI: 95% confidence interval.
n = 50 for both models (4 predictors each; subjects-per-variable = 12.5). AMA, age, sex (0 = male, 1 = female), and GMFCS group (0 = ambulatory [I–III], 1 = non-ambulatory [IV–V]) were entered simultaneously as independent variables; diaphragmatic thickness (inspiratory or expiratory, mm) was the dependent variable in each model. All variance inflation factors (VIF) were <1.4, indicating no problematic collinearity.
